# Supplementary material for: Human emotional odours influence horses’ behaviour and physiology
Source: PLoS One. 2026 Jan 14;21(1):e0337948. doi: 10.1371/journal.pone.0337948 (PMC12803465; doi:10.1371/journal.pone.0337948)

Human emotional odours influence horses' behaviour and physiology

Plotine Jardat[1,2¤], Alexandra Destrez[3], Fabrice Damon[3], Noa Tanguy-Guillo[2], Anne-Lyse Lainé[2], Céline Parias[2], Fabrice Reigner[4], Vitor H.B. Ferreira[2], Ludovic Calandreau[2], Léa Lansade[2¤]

[1] Institut Français du Cheval et de l'Equitation, Pôle développement, innovation et recherche, Nouzilly - France

[2]INRAE, CNRS, Université de Tours, PRC, 37380, Nouzilly, France

[3]Development of Olfactory Communication and Cognition Laboratory, Centre des Sciences du Goût et de l'Alimentation, Institut Agro Dijon, CNRS, Université de Bourgogne-Franche-Comté, Inrae, Dijon, France.

[4]UEPAO, INRAE, F-37380 Nouzilly, France

¤Corresponding authors – plotine.jardat@ifce.fr ; lea.lansade@inrae.fr

SUPPLEMENTARY INFORMATION

Table S1: Selection of the odour samples

Participant number	Sex	Followed hygienic and dietary instruction	Rating of feeling fearful during the Fear video	Rating of feeling joyful during the Joy video	Samples selected for experiment
1	F	yes	2	7	no
2	M	yes	3	5	no
3	F	yes	3	7	no
4	F	yes	6	3	yes
5	F	yes	3	7	no
6	F	yes	4	6	no
7	F	yes	7	5	yes
8	F	yes	4	4	no
9	M	yes	2	5	no
10	M	yes	4	5	no
11	F	yes	6	5	yes
12	F	no	4	6	no
13	F	yes	6	6	yes
14	F	yes	6	6	yes
15	F	yes	3	6	no
16	F	yes	5	6	yes
17	M	yes	3	3	no
18	F	yes	5	5	yes
19	F	yes	5	5	yes
20	F	no	1	5	no
21	M	yes	1	4	no
22	F	yes	6	5	yes
23	M	yes	6	6	yes
24	M	yes	5	3	yes
25	F	yes	5	6	yes
26	F	yes	5	6	yes
27	F	yes	5	6	yes
28	F	no	4	5	no
29	M	yes	4	7	no
30	F	yes	4	x	no

Samples from participants who had not followed the hygienic and dietary instructions were excluded. Among the remaining samples, those form the participants who had felt the most fearful during the fear video and joyful during the joy video were selected.

Table S2: Number of horses whose heart rate data were included in the analysis for each test. The initial group sizes were n=14 for the Joy group and the Fear group and n=15 for the Control group

Test	Joy group	Control group	Fear group
Human approach	13	13	13
Suddenness	10	10	11
Novel object	9	12	13

Table S3: Models used for the analysis of behavioural variables and results of their comparison to the null model (° p≤0.1, * p≤0.05, *** p≤0.001)**.**

**Test**	**Response variable (y)**	**Family**	**Model formula**	**χ^2**	**DF**	***p* value**
Grooming	Number of touching the experimenter	Generalized Poisson	y ~ Group	5.59	2	0.061°
Human approach	Number of touching the experimenter	Generalized Poisson	y ~ Group	7.27	2	0.026*
Suddenness	Intensity of startle	Gaussian	y ~ Group	9.02	2	0.011*
Novel object	Number of gazes	Poisson	y ~ Group	7.09	2	0.033*
	Number of contacts	Generalized Poisson	y ~ Group	6.22	2	0.045*
PCA	F1	Gaussian	y ~ Group	18.68	2	<0.001***
	F2	Gaussian	y ~ Group	0.38	2	0.83

Table S4: Models used for the analysis of physiological variables and results of their comparison to the null model (* p≤0.05)**.**

**Test**	**Response variable (y)**	**Family**	**Model formula**	**χ^2**	**DF**	***p* value**
Human approach	Mean heart rate	Gaussian	y ~ Group	0.57	2	0.75
	Maximum heart rate	Gaussian	y ~ Group	0.73	2	0.69
Suddenness	Mean heart rate	Gaussian	y ~ Group	1.79	2	0.41
	Maximum heart rate	Gaussian	y ~ Group	9.37	2	0.014*
Novel object	Mean heart rate	Gaussian	y ~ Group	2.71	2	0.26
	Maximum heart rate	Gaussian	y ~ Group	1.51	2	0.47
All tests	Variation in cortisol level	Gaussian	Y ~ Group	0.99	2	0.61

Table S5: Descriptive statistics and effect sizes for the behavioral and physiological variables. Effect sizes are given as the rate ratios (RR) or Cohen's d (d), as appropriate for each variable.

Test	Response variable (y)	Condition	Mean±s.e.	Effect size (mean±se)	
Grooming	Number of touching the experimenter	Joy	3.36±2.84		RR=1.18±0.28
		Neutral	2.80±2.27		RR=2.79±0.21
		Fear	1.43±1.40		RR=0.54±0.35
Human approach	Number of touching the experimenter	Joy	7.79±4.64		RR=1.11±0.20
		Neutral	6.60±3.20		RR=6.83±0.15
		Fear	4.14±2.93		RR=0.60±0.24
	Mean heart rate	Joy	58.64±10.16	Joy-Neutral	d=-0.22±0.39
		Neutral	61.67±15.12	Neutral-Fear	d=-0.062±0.39
		Fear	62.52±16.66	Joy-Fear	d=-0.28±0.39
	Maximum heart rate	Joy	110.92±34.56	Joy-Neutral	d=0.086±0.39
		Neutral	107.62±43.23	Neutral-Fear	d=-0.32±0.34
		Fear	120.13±41.93	Joy-Fear	d=-0.24±0.39
Suddenness	Intensity of startle	Joy	1.39±0.49	Joy-Neutral	d=0.326±0.37
		Neutral	1.23±0.42	Neutral-Fear	d=-1.20±-0.40
		Fear	1.82±0.61	Joy-Fear	d=-0.88±0.39
	Mean heart rate	Joy	76.77±16.91	Joy-Neutral	d=0.029±0.45
		Neutral	76.36±12.63	Neutral-Fear	d=-0.52±0.44
		Fear	83.72±14.57	Joy-Fear	d=-0.49±0.44
	Maximum heart rate	Joy	129.30±28.54	Joy-Neutral	d=0.14±0.45
		Neutral	125.03±20.55	Neutral-Fear	d=-1.31±0.47
		Fear	163.85±40.23	Joy-Fear	d=1.16±0.47
Novel object	Number of gazes	Joy	5.21±2.49		RR=0.90±0.16
		Neutral	5.80±2.96		RR=5.80±0.11
		Fear	7.64±3.43		RR=1.32±0.14
	Number of contacts	Joy	2.07±2.62		RR=1.12±0.73
		Neutral	1.53±1.73		RR=1.47±0.65
		Fear	0.36±0.63		RR=0.27±0.87
	Mean heart rate	Joy	66.47±15.72	Joy-Neutral	d=-0.73±0.45
		Neutral	78.03±14.12	Neutral-Fear	d=0.14±0.40
		Fear	75.89±19.04	Joy-Fear	d=-0.60±0.44
	Maximum heart rate	Joy	118.08±29.91	Joy-Neutral	d=-0.50±0.45
		Neutral	131.87±33.51	Neutral-Fear	d=0.031±0.40
		Fear	131.01±23.21	Joy-Fear	d=-0.47±0.44
PCA	F1	Joy	0.72±1.36	Joy-Neutral	d=0.32±0.37
		Neutral	0.38±1.21	Neutral-Fear	d=1.40±0.40
		Fear	-1.13±0.68	Joy-Fear	d=1.71±0.43
	F2	Joy	-0.077±1.43	Joy-Neutral	d=-0.21±0.37
		Neutral	0.15±1.04	Neutral-Fear	d=0.22±0.37
		Fear	-0.089±0.92	Joy-Fear	d=0.011±0.38
All tests	Variation in cortisol level	Joy	0.86±1.79	Joy-Neutral	d=-0.20±0.27
		Neutral	1.18±1.76	Neutral-Fear	d=0.25±0.27
		Fear	0.78±1.73	Joy-Fear	d=0.052±0.27

a

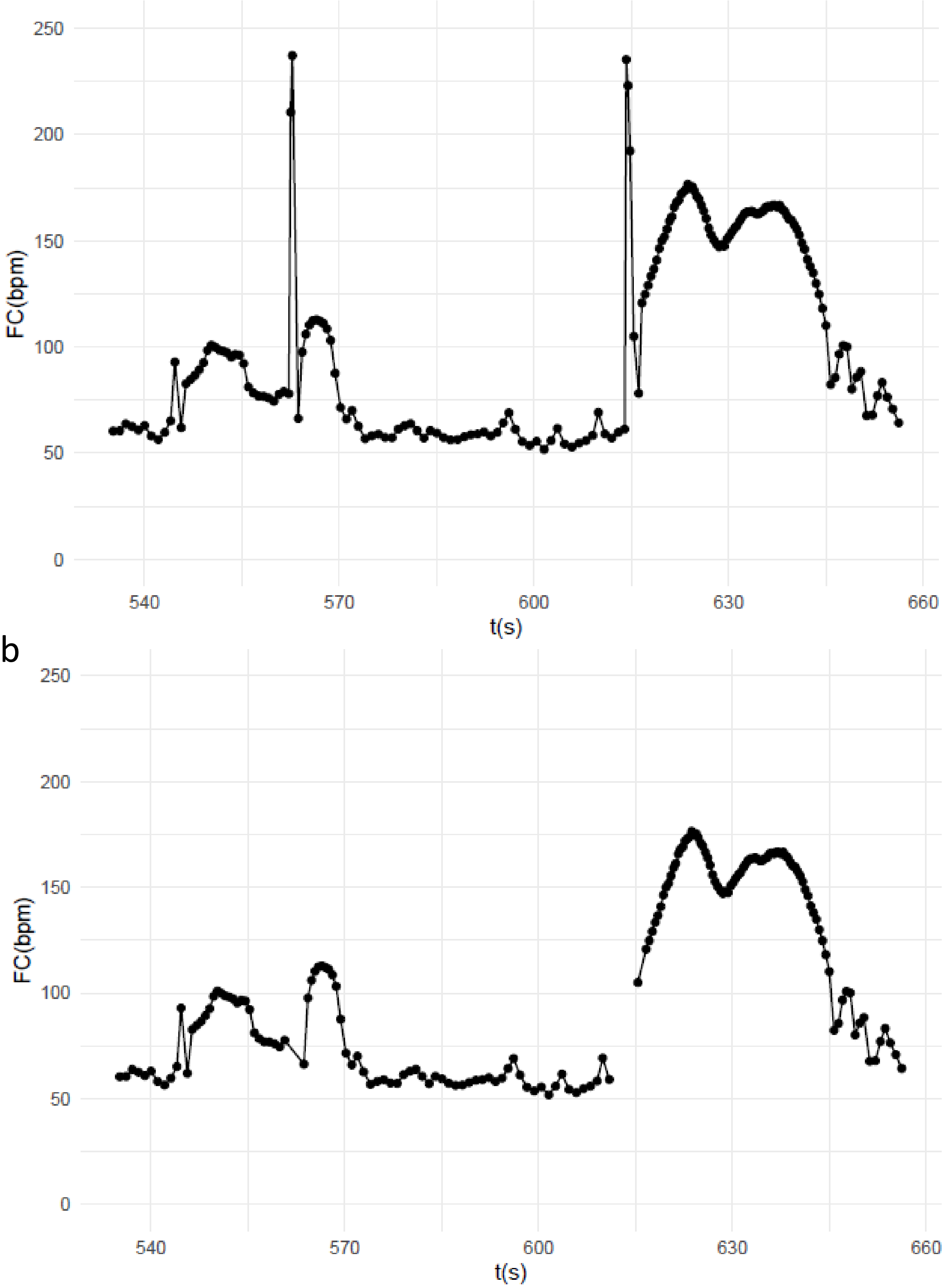

<u>Figure S1</u>: Example of heart rate data before (a) and after (b) clearing up to cut artefactual beats. The method used was examining each data point; if it had a difference of more than 35 bpm with the previous point, it was considered artefactual and removed from the data set and replaced by a missing value (NA).

<u>Figure S2</u>: Correlations between the behavioural variables

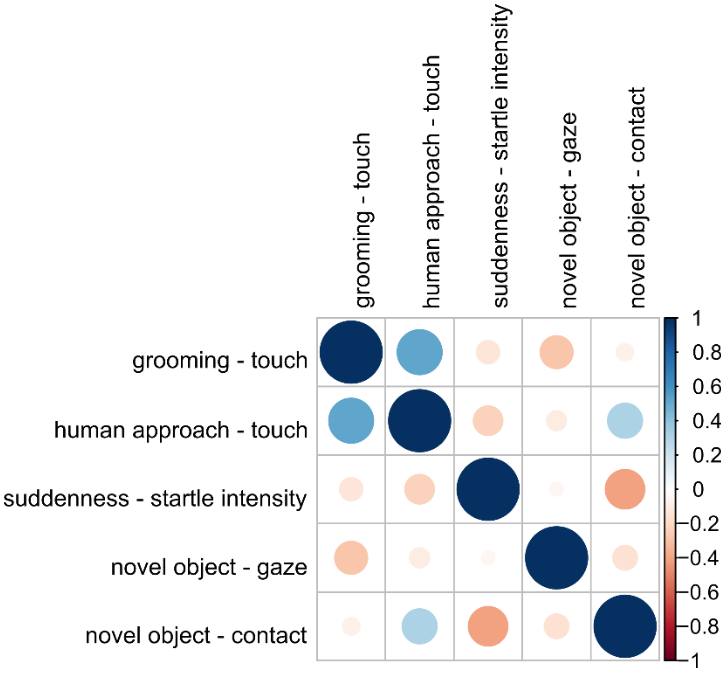

Supplement: S1 File — (PDF) [file pone.0337948.s001.pdf]
