# Supplementary material for: Human emotional odours influence horses’ behaviour and physiology
Source: PLoS One. 2026 Jan 14;21(1):e0337948. doi: 10.1371/journal.pone.0337948 (PMC12803465; doi:10.1371/journal.pone.0337948)
Supplement: Attachment — Submitted filename: Response to reviewers.docx [file pone.0337948.s003.docx]

Response to reviewers

# Editor comments

I have consulted with two researchers whose expertise closely aligns with your manuscript topic. Both reviewers acknowledge that your study is innovative and has the potential to enhance the field of human-animal interaction research. However, Reviewer 2 raises important concerns about the transparency of your methods and limitations, as well as your interpretations of the results (that do not align with your actual findings). They also encourage you to consider the practical applications of your research in greater depth. Meanwhile, Reviewer 1 has provided some minor grammatical suggestions that can be addressed during your revision process to incorporate their feedback.

*Dear editor,*

*Thank you for your comments on our manuscript. We have now revised the article, answering all the comments made by the two reviewers. Precisions have been added in the methods section and a new paragraph stating the limitations and perspectives of our study has been added in the discussion. Practical implications have also been added in accordance with the suggestions of reviewer 2. Moreover, we carefully revised the grammatical issues raised by reviewer 1 and the whole manuscript was screened for any additional language errors. Last, following the reviewers’ comments, the statistical analysis was adjusted, with minor impacts on the results and no impact on our conclusions (see the manuscript with track changes where the differences are highlighted).*

*We hope that these modifications will satisfy you and the two reviewers and that our manuscript is now suitable for publication in your esteemed journal.*

# Reviewer 1

I recommend publication of the manuscript after minor revision

*Thank you for taking the time to revise our manuscript, for your kind comments and for this recommendation. We modified the article following your remarks.*

Comments
The text needs some improvement. Some of the mistakes are mentioned below.

*Thank you for your vigilance. We have carefully revised the mentioned sentences and screened the whole manuscript for any other language error.*

Line 113 Human axillary sweat odour was collected from 30 adult participants. Were all participants women? If yes, mention it in the text. If no, mention how men and women were distributed. (I assume men and women smell different, especially in frightening situations.)

*Thank you for this question. The number of men and women is now mentioned in the methods section.*

*Line 115: “*Human axillary sweat odour was collected from 30 adult participants (8 males and 22 females) who volunteered to take part in the experiment.*”*

*Moreover, we agree, that sex, as well as other individual characteristics such as age, genetics, etc, could influence the smells of odor samples. As we used samples from each donor in each condition (fear and joy), the impact on our results should be limited. However, we have now included the identity of the donor in the statistical analysis to take this into account (with minor impact on the results).*

*Line 276 and 294: “*Odour donor identity was added as a random factor to account for individual factors potentially influencing emotional odours (sex, age, genetics, etc).*”*

*We also added a discussion about this in the new Limitations and Persepctives paragraph.*

*Line 454: “*Moreover, previous studies on human body odours revealed potential differences in nature and quantity of emotional body odours produced by men and women [81,82], and other parameters such as genetics, age or physical condition are also known to influence body odours [83–85]. Although donor identity was included as a random factor in our study to account for such potential variations, it would be interesting to determine in further studies the influence of these parameters on emotional communication through odours in human-animal interactions.*”*

Line 186-187 Change ‘second assistant were blind to the condition’ to ‘second assistant was blind to the condition’

*We meant that two people were blind to the condition: the second assistant and the experimenter. We modified the sentence to make this clearer.*

*Line 192: “*Only one of these assistants (hereby, the first assistant) was aware of which group the horse belonged to (Joy, Control or Fear), while the experimenter and the second assistant were both blind to the condition.*”*

Line 193 Change ‘in’ to ‘of’

*Thank you, this was modified according to your suggestion.*

Line 221 Change ‘were’ to ‘was’

*We meant that two behaviors were recorded, we modified the sentence to make this clearer.*

*Line 227:* “Two behaviours of the horse were recorded as measures of her fear toward the object [39,45]: the number of gazes at the object and the number of contacts the horse made with the object (touching the object with the nose or another body part).”

Line 252 Change ‘were’ to ‘was’
Line 285 Change ‘an chi-square test’ to ‘a chi-square test’
Line 357 Change ‘on’ to ‘of’

*Thank you, these sentences were modified according to your suggestions.*

Line 395-389 The sentence is a bit long. Change to ‘However, results indicate a gradient in horses’ reactions across the Joy, Control, and Fear groups, as depicted in graphical representations (Fig3a-d&f); and the detection of significant differences between the Fear group and the Joy group but not between the Fear group and the Control group. This result suggests that horses’ reaction to the control odours was not equivalent to that to that of the odour from the joy context.’

*Thank you for this suggestion. This sentence has now been removed from the manuscript following the comments of reviewer 2.*

Line 404 Change sentence ‘…suggests physiological responses to interspecific fear-related odours do occur,’ to ‘…suggests that physiological responses to interspecific fear-related odours do occur,’

*Thank you for this suggestion, we modified the sentence accordingly (this sentence is now part of another section following the comments of reviewer 2).*

*Line 428:* “On the other hand, increased maximum heart rate during the suddenness test suggests that physiological responses to interspecific fear-related odours do occur, which is in line with the function of emotions to recruit appropriate physiological resources in response to a stimulus, supporting survival [79].”

# Reviewer 2

Dear Authors,
This study addresses important and increasingly relevant topic in animal cognition and human–animal interaction, investigating the role of human emotional chemosignals in interspecific communication with horses.
Overall, the study is well-conceived and conducted, and most results support the authors’ hypotheses, providing novel perspectives on cross-species emotional transmission. However, several aspects require substantial clarification and revision before the manuscript can be considered for publication.

*Thank you for taking the time to revise our manuscript and providing useful comments to improve it. Please find below our answers to all your remarks. We hope that the major modifications we implemented now make our manuscript suitable for publication.*

Major Revisions Required

1. Clarification on Horses’ Responses to Joy Odours
The discussion states that horses showed a "gradient" of responses to joy, control, and fear odours, suggesting that control was not equivalent to joy. However, post hoc tests for individual behaviours (Table 2) do not show significant differences between Joy and Control in most variables, nor between Joy and Fear in some cases (e.g., startle intensity, number of contacts with the novel object). Request: Please revise the language regarding Joy odours. While the absence of significant differences is a valid result and the recommendation of future habituation–discrimination studies is appropriate, the discussion should be more cautious to avoid implying differences where statistical support is lacking. Consider offering a deeper explanation for the lack of clear behavioural response to Joy odours, possibly discussing the lower salience of positive chemosignals or the complexity of “joy” as an interspecific cue compared to “fear”.

*Thank you for this comment. We agree that the lack of significant differences in post hoc tests between Joy and Control, nor between Joy and Fear un some cases, make our conclusions on the differences between these odors weak. We removed the sentence about graphical differences and discussed the possible lower salience or complexity of positive chemosignals.*

*Line 418: “*However, positive chemosignals may be less salient than negative ones, or “joy” may have been too complex as an interspecific cue compared to “fear”, to trigger observable differences in behaviour and physiology in the present study.*”*

2. Cortisol Limitations
The lack of significant differences in cortisol levels is an important limitation. Request: While the discussion acknowledges possible reasons (circadian rhythms, short testing duration), this limitation should be more explicitly integrated—either in the Conclusion of the abstract or in a dedicated “Limitations” paragraph within the Discussion. This would help readers properly interpret the scope of the physiological responses. Increasing the sampling period, as suggested, is a valuable recommendation for future studies.

*Thank you for this remark. We added a discussion about the lack of significant differences in the cortisol analysis in a new Limitations and Perspectives paragraph (other issues are also discussed in this paragraph).*

*Line 424*: “In this study, no significant differences in cortisol level variations were observed, which limits the range of physiological consequences of human emotional odours on horses’ physiology. These results could be due to circadian rhythms inducing marked variability [55], or to this parameter varying over longer periods than our tests allowed (less than an hour per horse). Therefore, it would be interesting to repeat this investigation over more extended periods [77,78].”

3. Figure and Table Presentation
Figure 3: The caption is clear and the inclusion of individual data points is helpful. However, for some subplots (e.g., 3a, 3c), overlapping points make individual data hard to distinguish. Consider jittering or transparency to improve readability.

*Thank you for this suggestion, we added jittering and transparency to improve readability of this graph, all individual points should now be visible.*

Table 2: For post hoc tests, consider also providing the overall F or χ² statistic of the full GLMM before the pairwise comparisons, to give readers an overview of the group effect before detailing pairwise contrasts. Currently, only the χ² from the null vs. full model is shown.

*We are sorry that the tables were not clear enough. The χ² numbers provided in the main manuscript are indeed those of the full GLMM before the pairwise comparisons, while the χ² numbers for the null vs. full models are given in Supplementary Information. We have now made it clearer in the text and tables.*

*E.g., line 298: “*The models were compared to the null model via likelihood-ratio (chi-square) tests (results of these tests are given in Supplementary Information - Table S3).”

*And line 319:*

**Table 2: Results of the tests performed for each behavioural variable**

| **Test** | **Variable** | **Overall group effect** | | | **Post-hoc tests** | | |  |  |  |
| --- | --- | --- | --- | --- | --- | --- | --- | --- | --- | --- |
|  |  | Chi-square test | | Df |  |  |  |  |  |  |
| Grooming | Number of touching the experimenter | χ²=5.24 | **p=0.072°** | 2 | Joy-Fear | Z=2.26 | **p=0.062°** | | |  |
|  |  |  |  |  | Joy-Control | Z=0.57 | p=0.84 | | |  |
|  |  |  |  |  | Control-Fear | Z=1.76 | p=0.18 | | |  |
| Human approach | Number of touching the experimenter | χ²=6.91 | **p=0.032*** | 2 | Joy-Fear | Z=2.55 | **p=0.029*** | | |  |
|  |  |  |  |  | Joy-Control | Z=0.52 | p=0.86 | | |  |
|  |  |  |  |  | Control-Fear | Z=2.12 | **p=0.086°** | | |  |
| Suddenness | Intensity of startle | χ²=11.04 | **p=0.004**** | 2 | Joy-Fear | t=-2.31 | **p=0.066°** | | |  |
|  |  |  |  |  | Joy-Control | t=0.88 | p=0.66 | | |  |
|  |  |  |  |  | Control-Fear | t=-3.23 | **p=0.0071**** | | |  |
| Novel object | Number of gazes | χ²=7.20 | **p=0.027*** | 2 | Joy-Fear | Z=2.52 | **p=0.032*** | | |  |
|  |  |  |  |  | Joy-Control | Z=-0.67 | p=0.78 | | |  |
|  |  |  |  |  | Control-Fear | Z=-1.91 | p=0.14 | | |  |
|  | Number of contacts | χ²=5.84 | **p=0.054°** | 2 | Joy-Fear | Z=2.13 | **p=0.043*°** | | |  |
|  |  |  |  |  | Joy-Control | Z=0.26 | p=0.96 | | |  |
|  |  |  |  |  | Control-Fear | Z=1.98 | p=0.12 | | |  |
| PCA | F1 | χ²=23.4 | **p<0.001***** | 2 | Joy-Fear | t=4.54 | **p=0.0002***** | | |  |
|  |  |  |  |  | Joy-Control | t=0.85 | p=0.67 | | |  |
|  |  |  |  |  | Control-Fear | t=3.76 | **p=0.0016**** | | |  |
|  | F2 | χ²=0.45 | p=0.80 | 2 |  |  | | |  | |

° p≤0.1, * p≤0.05, ** p≤0.01, *** p≤0.001. Joy: horses exposed to cotton pads from humans watching a joyful video, Control: horses exposed to unused cotton pads, Fear: horses exposed to cotton pads from humans watching a horror video. Results of comparisons to the null model are available in Supplementary Information - Table S3.

4. Methodological and Interpretative Transparency
Sample of Human Donors: Clarify the criteria used to select 14 donors out of the original 30.

*Thank you for this suggestion. We modified the text to make this clearer.*

*Line 138:* “Among the remaining samples we selected the ones from 14 participants (2 males and 12 females) to match the number of horses included in the study (see Animals below). They were the 14 participants who experienced the most fear in the fear condition and the most joy in the joy condition (participants were sorted in ascending order according to their rating of feeling fearful in the fear condition and to their rating of feeling joyful in the joy condition; and the 14 best-ranking participants were selected – see Supplementary Information, Table S1).”

Animal Sample: Only Welsh mares (n=43) were tested. Explicitly discuss how this restricts generalization to other sexes, breeds, and ages. Audience Horse: The possible influence of an “audience horse” on focal horses’ behaviour is not mentioned as a limitation and should be acknowledged.

*Thank you for these suggestions. A discussion about the sample and one about the audience horse were added in the new Limitations and Perspectives paragraph.*

*Sample, line 434: “*Another limitation is the animal sample, which included only females of Welsh breed. Other breeds and sexes (males, geldings) could react differently to human emotional odours, although these factors have not been identified as significant regarding horse-human interactions nor horse olfactory perception in previous studies (e.g., [25,29,30,33,35,36]). Moreover, the horses were all aged 5 to 12 years, and it would be interesting to investigate the influence of age on the observed phenomenon through a larger age sample, as this factor has been shown to influence horses’ perception of human cues [80].”

*Audience horse, line 441:* “Regarding our experimental set-up, an audience horse was present for ethical reasons (preventing stress from social isolation), and in spite of all the precautions taken to limit her influence on the results (same audience horse across groups, habituated to the set-up and stimuli, her fence 1 m to the test box), consequences of her presence on the focal horse cannot be formally excluded. Further studies on horses’ response to human emotional odours in different contexts will be valuable to confirm the observed phenomenon.”

Heart Rate Data Quality: A large proportion of artefacts were removed, but the exact percentage of excluded data per condition is not reported. This threatens reliability and must be stated.

*Thank you for this comment. This is now discussed in the Limitations and Perspectives paragraph.*

*Line 428:* “On the other hand, increased maximum heart rate during the suddenness test suggests that physiological responses to interspecific fear-related odours do occur, which is in line with the function of emotions to recruit appropriate physiological resources in response to a stimulus, supporting survival [79]. However, the limited quality of heart rate data, and consequent data exclusion that was needed, encourage caution regarding these conclusions.”

5. Statistical Approach
The practice of considering p ≤ 0.1 as a “trend” without corrections for multiple comparisons could inflate false positives. Request: Justify this choice and consider using appropriate multiple comparison corrections (e.g., FDR, Bonferroni).

*Thank you for raising this issue. We agree that considering results with 0.05 ≤ p ≤ 0.1 and testing multiple variables could inflate false positives. However, corrections such as Bonferroni are debatable as they induce an inflation of false negatives and could lead to overlook interesting results (see Streiner, 2015.* Best (but oft-forgotten) practices: the multiple problems of multiplicity—whether and how to correct for many statistical tests. *The American Journal of Clinical Nutrition. doi:*[*10.3945/AJCN.115.113548*](https://doi.org/10.3945/AJCN.115.113548)*). As the topic of this study was novel and concerns a poorly understood phenomenon yet, we wanted to detect any possible effect of the samples on horses’ behavior and physiology; this is why we chose an approach with several variables and considered results with 0.05 ≤ p ≤ 0.1. One option could be to report these results differently if there are concerns about their interpretation. That said, readers should be able to assess the data for themselves, given that the p-value is clearly provided and individual data points are shown on graphs. Moreover, to strengthen our analysis of the effect of the samples on horses’ behavior, we conducted a Principal Component Analysis, which incorporates all behavioural variables and avoids multiple analyses. The results of this global analysis confirm a significant effect of the samples on horses’ behavior. Last, to avoid confusions carried by the word “trend”, we modified the manuscript to rather mention “marginally significant results”, as is the current recommendation for such results.*

*Line 265:* “All statistical analysis were performed using R 4.3.1 [47], and figures were generated using the packages *ggplot2* [48] and *factoextra* [49]. The significance threshold was set at α ≤ 0.05. As this was a pioneering study exploring a poorly understood phenomenon, we aspired to detect any effect of human emotional odors on horses’ behavior and physiology and also considered marginally significant results, with a criterion of p-values ≤ 0.1. Tukey’s method was employed to adjust p-values when performing multiple comparisons.”

6. Reporting of Effect Sizes and Variability
The abstract presents the results without effect sizes or statistical values, which reduces transparency. Request: Provide effect sizes for key behavioural variables and more detail on cortisol variability. A power analysis for the non-significant cortisol results would be valuable.

*Thank you for suggesting this. We added effect sizes in the abstract for key behavioural variables. All effect sizes are also available in Supplementary Information Table S5.*

*Line 28:* “More precisely, when exposed to fear-related odours, horses touched the human less in the human approach test (effect size: Rate Ratio(RR)=0.60±0.24), gazed more at the novel object (RR=1.32±0.14), and were more startled (startle intensity – Cohen’s d=-0.88±0.39; and maximum heart rate – Cohen’s d=1.16±0.47) by a sudden event.”

*More details about cortisol variability have been added in the Results section, and a post-hoc power analysis has been added to explore these results.*

*Line 304: “*Moreover, a post-hoc power analysis was performed for the evolution of cortisol level with the function *pwr.t.test()* from the package *pwr*.*”*

*Line 351: “*The variation in cortisol level showed marked variability in the sample (Mean±s.e., Joy: 0.86±1.79, Neutral: 1.18±1.76, Fear: 0.78±1.73) with no significant effect of the group (Table 4). Post-hoc power analysis revealed a power of 0.15, indicating that variability in the sample was too high considering the sample size to detect a significant effect in this study.*”*

7. Interpretation and Discussion
Some conclusions (e.g., emotional contagion) are overstated given the absence of chemical analyses of the odours and the lack of evidence on specific volatile compounds. Please tone down speculative statements and emphasize that these results are preliminary and context-specific.

*Thank you for these suggestions. The discussion has been modified to avoid implying the direct observation of emotional contagion of this study and tone down the conclusions.*

*Line 368: “*More than just a reaction to odours, there appears to be an analogy between the emotional state of the donor (human) and that of the receiver (horse). Indeed, the observed responses indicate a shift towards a more negative and higher arousal emotional state in horses, consistent with the emotional valence and arousal of the human emitters. Correspondence in emotional valence and arousal between an emitter and receiver corresponds to criteria of emotional contagion defined as the transmission of an emotional state between two individuals [56,57]. Therefore, our observations suggest that emotional contagion of fear through odours, previously observed among humans [58–60], could also occur interspecifically, although these results still need validation from independent studies.”

*A discussion about volatile compounds has also been added in the Limitations and Perspectives paragraph.*

*Line 447: “*Last, recent studies have started to identify chemical families and compounds that might be responsible for the emotional content of human body odours [15,16]. Therefore, in addition to selecting samples from human donors who were in the intended emotional states, which could be a source of bias, it would be interesting to analyze the chemical content of odours presented to horses or other animals in behavioural studies like the present.”

Explicitly expand the discussion of limitations: donor selection, restricted animal sample, data exclusion, absence of chemical identification of compounds. Clearly state that results may not generalize across breeds, sexes, or ages.

A new Limitations and Perspectives paragraph has been added to discuss all these parameters:

*Line 423: “*Limitations and perspectives

In this study, no significant differences in cortisol level variations were observed, which limits the range of physiological consequences of human emotional odours on horses’ physiology. These results could be due to circadian rhythms inducing marked variability [55], or to this parameter varying over longer periods than our tests allowed (less than an hour per horse). Therefore, it would be interesting to repeat this investigation over more extended periods [77,78]. On the other hand, increased maximum heart rate during the suddenness test suggests that physiological responses to interspecific fear-related odours do occur, which is in line with the function of emotions to recruit appropriate physiological resources in response to a stimulus, supporting survival [79]. However, the limited quality of heart rate data, and consequent data exclusion that was needed, encourage caution regarding these conclusions.

Another limitation is the animal sample, which included only females of Welsh breed. Other breeds and sexes (males, geldings) could react differently to human emotional odours, although these factors have not been identified as significant regarding horse-human interactions nor horse olfactory perception in previous studies (e.g., [25,29,30,33,35,36]). Moreover, the horses were all aged 5 to 12 years, and it would be interesting to investigate the influence of age on the observed phenomenon through a larger age sample, as this factor has been shown to influence horses’ perception of human cues [80].

Regarding our experimental set-up, an audience horse was present for ethical reasons (preventing stress from social isolation), and in spite of all the precautions taken to limit her influence on the results (same audience horse across groups, habituated to the set-up and stimuli, her fence 1 m to the test box), consequences of her presence on the focal horse cannot be formally excluded. Further studies on horses’ response to human emotional odours in different contexts will be valuable to confirm the observed phenomenon.

Last, recent studies have started to identify chemical families and compounds that might be responsible for the emotional content of human body odours [15,16]. Therefore, in addition to selecting samples from human donors who were in the intended emotional states, which could be a source of bias, it would be interesting to analyze the chemical content of odours presented to horses or other animals in behavioural studies like the present. Moreover, previous studies on human body odours revealed potential differences in nature and quantity of emotional body odours produced by men and women [81,82], and other parameters such as genetics, age or physical condition are also known to influence body odours [83–85]. Although donor identity was included as a random factor in our study to account for such potential variations, it would be interesting to determine in further studies the influence of these parameters on emotional communication through odours in human-animal interactions.

Overall, the relatively innovative character of this study and field of investigation makes our inferences subject to potential adjustments and precision from further studies conducted in other contexts.”

8. Practical Implications
The manuscript would benefit from a paragraph on the potential applied implications of these findings. For example:
Beyond their theoretical contribution to understanding interspecific emotional communication, these findings may have practical implications. In equine management and training contexts, human emotional states could directly influence horses’ behavioural and physiological responses, particularly when handlers experience fear or stress. This suggests that caretakers’ and riders’ emotional regulation could be an important component of equine welfare, safety, and training effectiveness. For instance, in equestrian sports, therapeutic riding programs, or clinical handling, human fear-related odours might inadvertently increase horses’ reactivity, potentially raising the risk of accidents or impairing human–horse interactions. Recognizing this pathway opens new avenues for designing training programs that integrate human emotional awareness, developing strategies to reduce stress transmission between humans and horses, and selecting or conditioning horses according to their sensitivity to human chemosignals.

*Thank you for suggesting this improvement, we implemented it in the manuscript.*

*Line 460:* “Practical Implications

Beyond their theoretical contribution to understanding interspecific emotional communication, these findings may have practical implications. In equine management and training contexts, human emotional states could directly influence horses’ behavioural and physiological responses, particularly when handlers experience fear or stress. This suggests that caretakers’ and riders’ emotional regulation could be an important component of equine welfare, safety, and training effectiveness. For instance, in equestrian sports, therapeutic riding programs, or clinical handling, human fear-related odours might inadvertently increase horses’ reactivity, potentially raising the risk of accidents or impairing human–horse interactions. Recognizing this pathway opens new avenues for designing training programs that integrate human emotional awareness, developing strategies to reduce stress transmission between humans and horses, and selecting or conditioning horses according to their sensitivity to human chemosignals.”

Minor Revisions and Editorial Suggestions

9. Abbreviations: Ensure that all abbreviations (PCA, GLMM, LMM, etc.) are defined at first use in the main text.

10. Citation Consistency: Check consistency between in-text citations and the reference list.

11. Language and Style: The manuscript is clearly written and professional. A final careful proofreading for minor typographical or grammatical errors is still recommended.

*Thank you for raising these points. We carefully revised the manuscript accordingly.*

Recommendation
This manuscript addresses an interesting research question with a solid methodological basis and valuable results, especially concerning fear odors. It is a valuable addition to the field of interspecific communication. Considering the strengths and the areas needing improvement, I recommend Major Revisions.

*Thank you for this recommendation. Following your comment, we made substantial changes to our manuscript and hope it now satisfies your requirements.*
